# Physical Activity, BMI, and Their Effects on University Students’ Quality of Life

**DOI:** 10.3390/healthcare13151880

**Published:** 2025-08-01

**Authors:** Ljubica Lalović, Danijela Živković, Anđela Đošić, Vanja Cicović, Borislav Cicović, Bojan Pavlović, Saša Pantelić

**Affiliations:** 1 Faculty of Physical Education and Sports, University of East Sarajevo, 71126 East Sarajevo, Bosnia and Herzegovina; milanoviclj85@gmail.com (L.L.); cicovicvanja9999@gmail.com (V.C.); borislav.cicovic@ffvis.com (B.C.); mfizikal@gmail.com (B.P.); 2 Faculty of Sport and Physical Education, University of Niš, 18000 Niš, Serbia; andjela.djosic88@gmail.com (A.Đ.); spantelic2002@yahoo.com (S.P.)

**Keywords:** physical activity, health behaviors, body composition, body mass index, university students

## Abstract

**Objectives:** The aim of this study was to examine the impact of physical activity levels and body mass index (BMI) on the quality of life among university students. **Methods:** The sample consisted of 495 students (176 males and 319 females). Physical activity was assessed using the International Physical Activity Questionnaire—Short Form (IPAQ-SF), while quality of life was measured using the WHOQOL-BREF questionnaire. Pearson’s correlation coefficient and regression analysis were employed to determine relationships and predictive influence. Data were analyzed using SPSS version 20, with the level of significance set at *p* < 0.05. **Results:** The results indicated that male students reported significantly higher levels of moderate and vigorous intensity physical activity compared to female students (*p* = 0.015 and *p* = 0.001, respectively), as well as higher scores in the physical health and social relationships domains of quality of life (*p* = 0.002 and *p* = 0.001, respectively). Both physical activity and BMI had a statistically significant impact on the physical health (*p* = 0.040 for males; *p* = 0.024 for females) and psychological health (*p* = 0.047 for males; *p* = 0.000 for females) domains. Specifically, moderate-intensity PA positively influenced physical health (β = 0.21, *p* = 0.005), while BMI was a predictor of psychological health in males (β = 0.18, *p* = 0.016). Among females, BMI negatively influenced physical health (β = −0.18, *p* = 0.002), and both low-intensity PA (β = 0.17, *p* = 0.002) and BMI (β = −0.21, *p* = 0.000) significantly affected psychological health. **Conclusions:** These findings underscore the importance of promoting diverse forms of physical activity and maintaining a healthy BMI in student populations, with consideration for gender-specific approaches to maximize quality of life outcomes.

## 1. Introduction

Regular physical activity contributes to the prevention of chronic non-communicable diseases such as cardiovascular diseases, cancer, and diabetes; reduces symptoms of depression and anxiety; improves cognitive abilities; and can enhance overall health [1,2]. In addition, physical activity contributes to strengthening the immune system [3] and reducing mental stress [4], making it a key component of a healthy lifestyle for the student population. Furthermore, regular physical activity improves sleep quality [5] and fitness parameters, including cardiorespiratory and muscular fitness, flexibility, and body composition [6], which makes students more resilient to physical challenges and contributes to their better performance. Moreover, physical activity positively impacts cognitive abilities, such as concentration, memory, and information processing speed [7], which can significantly contribute to their success in learning. A lack of physical activity, on the other hand, increases the risk of various health problems, which can negatively impact daily life and academic performance in the student population [8].

University life is a dynamic and pivotal stage of development, where students encounter numerous challenges and risks that strongly influence their health and daily habits. Changing living environments and increased responsibilities, as well as adapting to new learning methods and academic schedules, are just some of the sources of stress during this time [9]. These challenges often lead to the development of inadequate habits, which can negatively affect their overall health.

The World Health Organization [10] has provided recommendations on regular physical activity, which suggest a minimum of 150 min of moderate or vigorous physical activity per week. Following these recommendations for physical activity levels helps preserve and improve health by maintaining and reducing the decline of motor and functional abilities, which positively impacts the quality of life [11].

Although guidelines exist, many studies show that students’ engagement in physical activity is low. In the study by [12], a prevalence of 52% of insufficiently physically active students was recorded, while the research by Downs et al. [13] found that 53.1% of female students met the recommended level of physical activity.

Studies examining the level of physical activity in Bosnia and Herzegovina have demonstrated inconsistencies in the data. Doder et al. [8] indicate that the student population in Bosnia and Herzegovina is predominantly engaged in moderate-intensity physical activities at an adequate level, whereas other studies have concluded that physical activity is maintained at a satisfactory level [9]. Furthermore, female students have been found to be less physically active than their male counterparts [9].

Physical activity has a positive and significant impact on the quality of life of students, with a clear connection to various domains of quality of life [14]. These findings suggest that physical activity is a key factor in improving quality of life, while a low level of physical activity is often associated with an increased presence of psychosomatic disorders, motor function impairments, and reduced social functioning abilities [15]. For this reason, many researchers emphasize the importance of assessing the quality of life in the university student population [16]. Ge et al. [17] confirmed in their study that physical activity and sleep positively affect the quality of life in the student population, and similar results were obtained by Pekmezović et al. [18]. According to a review study by Taylor et al. [19], regular physical activity affects various aspects of students’ quality of life, although there is disagreement regarding the optimal intensity of exercise. Yet, despite this consensus, there is ongoing debate regarding which intensity level of physical activity yields the greatest benefit. While some findings emphasize the superior impact of moderate-to-vigorous activity [6,12], others argue that even light-intensity activity can significantly enhance psychological well-being [20]. This inconsistency suggests a need for more nuanced investigation into the dose–response relationship between physical activity and quality of life.

In addition to enhancing overall quality of life, participation in exercise programs has been found to positively impact body image satisfaction, which is a key indicator of psychological well-being.

Simultaneously, the role of Body Mass Index (BMI) in determining the health-related quality of life among university students has gained increasing attention; however, findings remain inconsistent. While a higher BMI is frequently associated with reduced quality of life—particularly in physical and social domains [13,14,21]—some studies suggest that this relationship may be mediated by factors such as gender, self-perception, and physical activity levels. Other studies, however, point to a direct association between physical activity and quality of life, independent of BMI [15,18,22,23]. Furthermore, the majority of existing research examines physical activity and BMI in isolation, without considering their combined effects or potential interactions. These inconsistencies may partly be attributed to differences in analytical methods as well as the specific characteristics of the studied populations. For example, research conducted in Iran has shown that men tend to have higher levels of physical activity and also higher BMI compared to women, and that BMI has a stronger impact on quality of life in men.

The university period represents a key developmental phase in the lives of young adults, during which habits with long-term health implications are established. As such, this life stage offers a final comprehensive opportunity for preventive action through the promotion of healthy lifestyle choices. The rationale behind this study is grounded in an identified gap within the existing literature—namely, the lack of research that simultaneously examines the impact of different intensities of physical activity and nutritional status (as reflected by BMI) on quality of life, while also considering gender differences and the multidimensional nature of well-being. This forms the foundation for the study’s research focus, which aims to systematically explore how variations in physical activity and body mass contribute to students’ subjective experiences of physical and mental well-being.

An additional layer of justification lies in the recognition that quality of life in men and women is shaped by distinct biological, psychological, and sociocultural factors, which in turn influence their behaviors related to physical activity and body weight. Hormonal differences, stress response mechanisms, and body image perceptions may lead to gender-specific patterns in how BMI and physical activity affect quality of life (Kuehner [19]). Therefore, consideration of gender differences is crucial for understanding the variability in the effects of these predictors. Neglecting such distinctions may result in less effective interventions, as universal approaches often fail to adequately meet the needs of both genders. By integrating these variables and emphasizing gender-specific dynamics, this study constitutes a well-reasoned effort to inform the development of more relevant and targeted health promotion strategies within the academic setting.

## 2. Materials and Methods

### 2.1. The Study Design and Procedures

The study employs a cross-sectional and correlational design to explore the associations between physical activity levels, body mass index (BMI), and various domains of quality of life in a university student population. The scope of the study is to examine the associations between physical activity levels, BMI, and various domains of quality of life among university students at a single point in time.

The study involved answering a questionnaire on PA levels and quality of life and assessing BMI.

Participants were also informed about the study, and the purpose and goals of the study were explained to them. The study participants were recruited from the faculties of the University of East Sarajevo and the University of Banja Luka, and participation in the study was voluntary.

The questionnaires were distributed and collected by faculty members from the Faculty of Sport and Physical Education, ensuring consistency and professionalism throughout the data collection process. Participants completed the questionnaires independently, without the presence of administrators during completion, to minimize external influences and maintain the objectivity of the data. Responses were entered directly into the questionnaire by participants, after which the obtained data were digitized for further analysis. There was no time limit imposed for completing the questionnaire, and participation was entirely voluntary, with participants free to withdraw from the study at any point. To promote honesty and impartiality, participants were informed that their responses would remain anonymous and that the collected data would be used solely for research purposes. Questionnaires that were incomplete were excluded from subsequent analyses.

Body weight and height measurements were taken after completing the questionnaires and were carried out by trained measurers. The study was approved by the Ministry of Education and Culture of the Republic of Srpska (No. 07.05/059-354-1/21 from 12 March 2021) and the Republic Pedagogical Institute of the Republic of Srpska (07/2.01/03-614-103/21 from 25 February 2021) and was conducted in accordance with the Helsinki Declaration and recommendations for research involving human subjects [24,25].

### 2.2. Sample of Participants

The population of students from which the sample was selected consisted of a total of 495 participants, including 176 male students (22.5 ± 2.8 years old, height 183.8 ± 8.0 cm, and weight 82.9 ± 14.9 kg) and 319 female students (21.6 ± 1.7 years old, height 168.9 ± 6.1 cm, and weight 62.1 ± 8.8 kg). The average recorded BMI value for male students was 24.3 ± 4.1, while for female students it was 21.7 ± 2.7.

The inclusion criteria for the study were as follows: the participants had to be active students (male and female) from any faculty of the University of East Sarajevo (Bosnia and Herzegovina); they had to have given their consent for voluntary participation in the research prior to the start of the study; they must not have any current health issues; and they should not have had any physical injuries in the past 6 months.

The exclusion criteria for the study were as follows: participants who were in the recovery phase from any acute illness or in the process of rehabilitation from injuries.

### 2.3. Sample Size

The sample size was determined using the G*Power 3.1 analysis program [26]. It was assumed that the effect size (f2) = 0.15, the alpha level = 0.05, and the power = 80% (0.80), so the estimated total sample size for each group was at least 85 participants per group. G*Power was used to determine the minimum required number of participants for each group. The influence of predictors on the criterion variable was assessed separately for each group, and the selected sample consisted of individuals who voluntarily agreed to participate in the study.

### 2.4. BMI

All measurements used to determine the characteristics of the sample (body mass, body height, and Body Mass Index—BMI) were carried out after the survey in accordance with the recommendations of Eston & Reilly [27]. Body height was measured using a Martin anthropometer (GPM, Bachenbülach, Switzerland) (measurement accuracy 0.1 cm). Body mass was measured using a digital scale (Omron BF511), and BMI is calculated as the ratio of body mass (kg)/body height (m^2^) [28].

### 2.5. Physical Activity

The level of PA was determined using the self-reported physical activity questionnaire—the International Physical Activity Questionnaire (IPAQ) Short Form [29]—whose reliability and validity have been established in various studies [30,31]. The short version of the IPAQ is designed to provide separate scores for each type of activity: (1) low-intensity PA, (2) moderate-intensity PA, and (3) high-intensity PA, and the assessment is made through seven questions. The questions focus on the time a person spent engaging in physical activity and resting (sitting, lying down) in the past seven days. To calculate the results for each domain of physical activity in relation to minutes per day, the metabolic equivalent (MET) was used. Total weekly MET-minutes (MET-min/week) were calculated by adding the MET-minutes for each level of PA intensity (low intensity = 3.3 MET; moderate intensity = 4.0 MET; high intensity = 8.0 MET).

### 2.6. Quality of Life

The quality of life of students was assessed using the short version of the World Health Organization Quality of Life Questionnaire (WHOQOL—BREF) [32]. A total of 26 questions were distributed across 4 domains: physical health, psychological health, social relationships, and environment, providing the possibility to calculate specific scores for each domain. The questions were presented on a five-point Likert scale, with the following response options: (1) not at all, (2) a little, (3) moderately, (4) very much, and (5) extremely. The higher the values, the better the quality of life. The reliability and validity of the WHOQOL-BREF questionnaire have been confirmed in various studies [33,34,35,36]. Cronbach’s alpha for the WHOQOL-BREF questionnaire in our study was 0.76.

### 2.7. Statistical Data Analysis

For each variable, basic descriptive statistics were calculated: mean and standard deviation (mean ± SD). Differences between groups were determined using the Student’s *t*-test. Correlations between physical activity levels, nutritional status, and quality of life were determined using Pearson’s correlation coefficient. The impact of physical activity and nutritional status on the quality of life (QOL) of the student population was assessed using regression analysis (enter method). Only participants with complete data for all measurement instruments were included in the statistical analysis. The normality of distribution was assessed using a standardized residuals plot, which indicated that the residuals followed a normal distribution. All data were processed using the SPSS 20.0 statistical software package (SPSS Inc., Chicago, IL, USA). The significance level was set at 0.05.

## 3. Results

The basic descriptive statistics parameters for the student population are presented in Table 1.

The results showed that male students have a higher level of PA and that they significantly differ from female students in moderate-intensity PA (*p* < 0.05) and vigorous-intensity PA (*p* < 0.01). Additionally, there are differences in BMI (*p* < 0.01), benefiting male students, as well as in certain domains for assessing quality of life (physical health *p* < 0.01; social relationships *p* < 0.01).

The cross-correlations (Pearson’s coefficient) between physical activity levels and BMI with quality of life domains for the sample of male and female students are presented in Table 2. The results showed that for male students, there are associations between moderate-intensity PA and physical health (*p* = 0.006); moderate-intensity PA and environmental (*p* = 0.040); and BMI and psychological health (*p* = 0.008). The cross-correlations between physical activity levels and BMI with quality of life for female students showed significant relationships between BMI and physical health (*p* = 0.001), BMI and psychological health (*p* = 0.000), BMI and environmental (*p* = 0.025), low-intensity PA and psychological health (*p* = 0.001), and high-intensity PA and social relationships (*p* = 0.008).

The regression analysis results (Table 3) showed that for both male and female students, the predictor had an impact on the criterion for physical health (*p* < 0.05, Sig = 0.040 for male students; *p* = 0.024 for female students) and psychological health (*p* < 0.05, Sig = 0.047 for male students; *p* < 0.01, Sig = 0.000 for female students).

**Figure 1:**
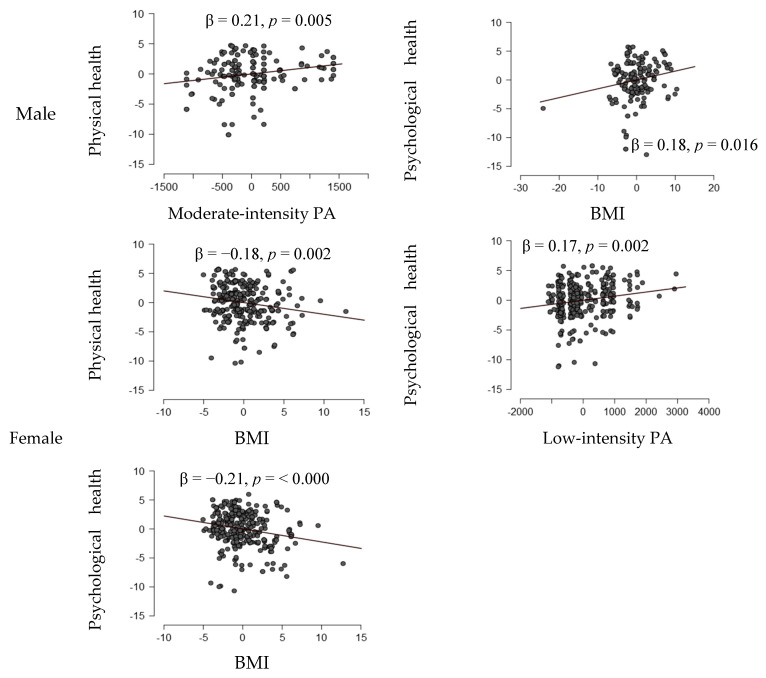
Partial impacts of individual variables on the criterion (standardized regression coefficients β).

The impact of individual variables of physical activity levels and BMI on the criterion was determined using the standardized regression coefficient and the obtained beta (β) value (Figure 1). The results of the standardized partial regression coefficients showed that for male students, the greatest impact on physical health is from moderate-intensity PA (β = 0.21, *p* = 0.005), while BMI has the greatest influence on psychological health (β = 0.18, *p* = 0.016). For female students, BMI has the greatest impact on physical health (β = −0.18, *p* = 0.002), while low-intensity PA and BMI have a significant impact on psychological health (β = 0.17, *p* = 0.002, β = −0.21, *p* = 0.000, respectively).

## 4. Discussion

The present study provides insight into the relationship between physical activity, BMI, and quality of life among university students. The descriptive results revealed lower physical activity levels compared to previous studies on similar populations [37,38,39], which may reflect the broader trend of declining physical activity post-COVID-19 [40]. Male students reported higher engagement in physical activity at all intensity levels, consistent with earlier findings [6,21]. Male students reported a higher quality of life compared to female students, particularly in the domains of physical health and social relationships. These results can be explained by the fact that male students are more involved in sports and social activities and have a more favorable response to physical and psychosocial challenges compared to female students [41]. Gender differences in quality-of-life predictors may also arise from differences in how health, physical appearance, and social roles are internalized. Physical activity may be more strongly linked to performance and achievement in men, while in women it may relate more to appearance and self-worth, shaped by cultural norms. These observed differences indicate that similar patterns of behavior may exert divergent impacts on quality of life contingent upon an individual’s gender. Male students often have different socialization patterns, which include team sports and group activities, contributing to the strengthening of the social relationships domain [42]. Active participation in sports or other group physical activities can improve the sense of belonging and provide social support. On average, male students may also have better fitness parameters, such as greater muscle mass and better cardiorespiratory fitness [43], which enables them to endure physical and mental challenges more easily. This may contribute to the perception of better physical and physical health and functionality [44]. While gender differences in physical activity and quality-of-life outcomes are well-documented, they may also reflect deeper psychological and sociocultural mechanisms. Female students, for example, may be more exposed to appearance-related pressures and internalize social expectations differently than male students, which could influence how they perceive and report well-being. Likewise, differences in coping strategies and health-related behaviors, shaped by gender norms, may affect the strength and direction of associations between variables such as BMI, physical activity, and quality of life. Therefore, stratified analyses are not only methodologically appropriate but also necessary to capture potentially divergent pathways through which quality-of-life outcomes are formed.

The results of the regression analysis show that moderate physical activity has an impact on the physical health domain in male students. Moderate-intensity physical activity was found to be a significant predictor of quality of life in the study by Slimani et al. [45], where a direct correlation was established between this level of physical activity and the physical domain of quality of life, among others, in individuals aged 18–30 years. This means that students who regularly engage in moderate-intensity activities experience improvements in their physical health, as moderate-intensity physical activity contributes to better health parameter values [46], as well as an overall sense of vitality [47], which together enhances the overall physical health quality of the student population.

BMI influences the psychological health domain in male students, meaning that students’ weight status affects their mental and emotional state, as confirmed by other studies [48,49]. Students with a BMI higher than normal may have lower self-esteem and body image [50], which can impact their psychological health. For example, students with a BMI within the recommended range may experience greater physical and mental vitality, which can contribute to a more positive psychological state [22]. On the other hand, lower BMI in male students may cause some students to experience stress or dissatisfaction with their appearance, negatively affecting their emotional balance and mental health [51]. Although the study did not investigate whether the student sample engages in organized sports, it is assumed that some students with higher BMI values are involved in sports or recreational activities that contribute to muscle building, thus having a higher BMI, which increases satisfaction with their physical appearance [22]. A higher BMI level does not necessarily correlate with a lower quality of life (QOL), especially in specific populations such as physically active students. BMI measures the ratio of body weight to height but does not distinguish between muscle mass and fat tissue. In individuals who are physically active, particularly in activities aimed at strength development and muscle building (e.g., recreational gym workouts, strength sports, martial arts), a higher BMI may reflect greater muscle mass, rather than excess body fat. A student with a BMI in the “higher” range (e.g., over 25) may be very satisfied with their physical appearance and health because they are muscular, physically fit, and active. Additionally, engaging in physical activity contributes to better health [1], stress reduction [52], and increased self-confidence [50], all of which can contribute to a higher QOL, despite higher BMI.

For female students, BMI has a statistically significant impact on physical health. BMI reflects the balance between energy intake and expenditure, which directly influences bodily functions. Overnutrition not only increases the risk of metabolic disorders, such as insulin resistance and type 2 diabetes, but also contributes to the development of hypertension, dyslipidemia, and increased visceral fat mass, which are key risk factors for cardiovascular diseases [53]. In addition to metabolic effects, a high BMI can have mechanical consequences, such as increased strain on the joints and spine, leading to chronic pain, reduced mobility, and an increased risk of injury [18]. This further limits the ability of female students to be physically active, creating a vicious cycle: a lack of physical activity leads to further increases in BMI, which further impairs physical health. A low BMI can be concerning, as it often indicates a lack of body mass, which may result from poor nutritional habits or the body’s inability to maintain muscle mass. The loss of muscle mass is linked to extended recovery periods from illness, slower healing of wounds, a reduction in resting metabolic rate, greater physical limitations, diminished quality of life, and increased healthcare costs [54].

The results showed that BMI also significantly impacts the psychological health of female students. This connection can be explained by the complex interactions between body mass and body image perception, which are often more pronounced in women. Society imposes strict beauty standards on women, with ideals of thinness being intensely promoted through the media, social networks, and advertisements. Such standards can create pressure on women to appear “perfect,” which increases the risk of body dissatisfaction, especially among those with higher BMI [55]. Female students with excess body weight often face dissatisfaction with their appearance [49], which can negatively affect their self-esteem [50]. Lower self-esteem can lead to reduced motivation for social interactions and greater withdrawal, further worsening psychological health [56]. Furthermore, a high BMI is associated with increased anxiety and a tendency toward depressive symptoms, which can stem from social stigmatization [57].

Among female students, BMI was significantly related to both physical and psychological health. These associations may reflect broader sociocultural and physiological influences, as higher BMI has been linked with reduced mobility and body dissatisfaction, particularly among young women [18,49,53,54,55,56,57]. Although hormonal and inflammatory mechanisms have been suggested in the literature [58,59], the present study did not investigate these factors and cannot draw conclusions in this regard.

The results obtained in our study show that low-intensity physical activity has a positive effect on the psychological health of female students. Other studies have shown that low-intensity physical activities reduce stress [60] and contribute to better emotional well-being [61], without causing excessive physical strain or fatigue. Activities such as walking or light exercise help regulate mood and improve feelings of calmness [60], which positively impacts psychological health. Additionally, engaging in low-intensity physical activity can increase feelings of satisfaction and self-confidence, while simultaneously contributing to improved overall well-being in women without excessive physical effort that could lead to counterproductive effects [20].

## 5. Conclusions

Quality of life is a multidimensional concept that encompasses physical, psychological, and social health, and all of these aspects are significantly influenced by the level of physical activity and BMI. In modern society, physical activity is increasingly recognized as an important preventive strategy for improving overall health and reducing the risk of chronic diseases in the student population. The results of this study indicate a significant impact of different levels of physical activity and BMI on students’ quality of life. Moderate physical activity significantly contributes to the improvement of physical and psychological health, positively influencing their overall quality of life. In female students, low-intensity physical activity and BMI affect psychological health.

Low- and moderate-intensity physical activity proves to be a key predictor of quality of life, contributing to both physical and psychological health. At the same time, BMI, as an important indicator of nutrition, plays a significant role in shaping quality of life in both genders, with the potential to positively or negatively impact various dimensions of health depending on the values and individual status of the person.

These results highlight the importance of implementing physical activity of appropriate intensity while considering gender-specific factors, as well as maintaining a healthy body weight, in order to improve the quality of life in the student population.

## 6. Limitations

While this study offers meaningful insights into the relationship between physical activity and quality of life in university students, several limitations should be considered when interpreting the findings. First, the study used a cross-sectional design, which allows for the identification of associations but does not permit conclusions regarding causality. Longitudinal studies would be needed to better understand the direction and long-term implications of these relationships. Second, while the sample size was adequate and based on power analysis, it consisted solely of students from a single university, which may limit the generalizability of the findings to other academic or cultural settings. Finally, although the study followed standardized procedures for data collection and measurement, including trained personnel and validated tools, certain external factors (e.g., academic workload, stress levels, or lifestyle differences) that could influence both physical activity and quality of life were not controlled for in the analysis. Despite these limitations, the study provides a valuable contribution to understanding the role of physical activity in shaping student well-being and highlights the importance of encouraging active lifestyles in university populations.

## Figures and Tables

**Table 1:** Descriptive statistics and differences between men and women.

	Men (n = 176)		Women (n = 319)		t	*p*
	Mean ± SD	[95% CI]	Mean ± SD	[95% CI]		
Low-intensity PA	1260.72 ± 785.41	[1148.45–1379.07]	1239.04 ± 752.74	[1156.22–1327.51]	0.29	NS
Moderate-intensity PA	1091.36 ±567.08	[1015.20–1177.55]	963.18 ± 548.56	[904.46–1022.05]	2.43	0.015 *
Vigorous-intensity PA	1108.56 ± 735.38	[1000.57–1222.44]	893.05 ± 607.01	[827.13–961.05]	3.31	<0.001 **
BMI (kg/m^2^)	24.30 ± 4.05	[23.69–24.88]	21.74 ± 2.71	[21.44–22.06]	7.48	<0.001 **
Physical health	30.28 ± 2.86	[29.86–30.68]	29.42 ± 3.08	[29.07–29.77]	3.11	0.002 **
Psychological health	24.56 ± 3.42	[24.06–25.06]	24.48 ± 2.96	[24.15–24.80]	0.26	NS
Social relationships	8.10 ± 1.35	[7.90–8.30]	8.50 ± 1.14	[8.37–8.63]	−3.30	<0.001 **
Environmental	31.41 ± 4.01	[30.83–32.02]	30.95 ± 4.49	[30.47–31.45]	1.17	NS

PA—physical activity (2 METs); Mean—mean value; SD—standard deviation; CI—confidence interval; *p*—the level of significance; NS—not significant; ** *p* < 0.01, * *p* < 0.05.

**Table 2:** Relationships between PA and BMI with QOL (Pearson’s r).

		Physical Health	Psychological Health	Social Relationships	Environmental
		r	r	r	r
Men	Low-intensity PA	0.092	0.109	0.053	0.086
	Moderate-intensity PA	0.190 **	0.087	0.045	0.132 *
	High-intensity PA	0.028	−0.036	0.033	0.002
	BMI	0.017	0.180 *	0.069	−0.058
Women	Low-intensity PA	0.032	0.170 **	−0.009	0.007
	Moderate-intensity PA	−0.025	−0.021	0.000	−0.086
	High-intensity PA	0.045	0.003	0.134 **	−0.045
	BMI	−0.173 **	−0.201 **	0.015	−0.110 *

Legend: ** Correlation is significant at the 0.01 level; * Correlation is significant at the 0.05 level.

**Table 3:** Regression analysis.

	Male			
	R	R Square	F	*p*
Physical health	0.238	0.06	2.56	0.040 *
Psychological health	0.233	0.05	2.46	0.047 *
Social relationships	0.116	0.02	0.58	NS
Environmental	0.183	0.03	1.48	NS
	Female			
	R	R Square	F	Sig
Physical health	0.187	0.04	2.85	0.024 *
Psychological health	0.266	0.07	5.99	<0.000 **
Social relationships	0.135	0.02	1.44	NS
Environmental	0.143	0.02	1.64	NS

Legend: NS—not significant; *p*—the level of significance; * *p* < 0.05, ** *p* < 0.01

## Data Availability

The data presented in this study are available on request from the corresponding author.
